# Botanical Description of British Plants

**Published:** 1804-08-01

**Authors:** 


### *Botanical Description of British Plants.*

( Continued from our last, pp. 28—37. )

### Class V. Pentandria.

#### Pentandria, Monogynia.

1. Lithospermum. *L. arvense. L. Vulgare. Anchusa degener.*

*Ang.* Bastard gromill, salern, corn gromwell, painting root, bastard Alkanet.

*Gen. Desc.* Bloss. funnel-shaped, tube long, slender, open, without valves at the mouth. cal. 5-div. nuts 4, very hard, imperforated.

*Spec. Desc.* *Seeds* wrinkled, generally 3 perfect with 1 abortive. *Bloss.* hardly longer than the cup, white. *Roots* crimson red, *corn fields.* Bloss. May, June.

*Use.* The girls in the north of Europe use the juice of the bright red roots of this plant for the purpose of painting their faces on days of festivity. The bark of the root tinges wax and oil of a beautiful red colour, similar to that obtained from the foreign alkanet root, which is sold in the shops. Sheep and goats eat it; cows are not fond of it; horses and swine refuse it.

2. Cynoglossum. *C. officinale. C. vulgare.*

*Ang.* Great hound's tongue.

*Gen. Desc.* Bloss. funnel shaped, mouth closed by projecting valves; nuts 4; depressed, fixed to the style by the inner side only, imperforated

*Spec.*

*Spec. Desc.* *Stamens* shorter than the blossom. *Leaves* broad strap-spear-shaped, sitting, cottony; the whole plant downy; cal. segments oblong egg-shaped. *Bloss.* marone colour; *valves* fringed. *Road sides, rubbish.* Bloss. June.

*Use.* It is succulent and somewhat mucilaginous, especially the root, which, for medicinal purposes, is preferred to the leaves. It is reported to be *deleterious*, and the dingy lurid appearance of the leaves, peculiar to poisonous shrubs of the narcotic kind, favours the opinion; nor are facts wanting to confirm it. A whole family, eating it by mistake for comfrey, severely experienced its ill effects. (*See Morrison Hist. Oxon. III.* 450.) Experience has not yet determined how far it may be employed as a medicine. Dr. Hulse is said to have prescribed a decoction of roots internally, together with a poultice of them, to *scrophulous tumours* with safety and advantage (*Ray, l. c.*). For its use in *coughs, hæmoptysis, diarrhœa, dysentery*, see *Schreckius, Diss. de Cynoglosso*, and *Woodville.* Both the root and the leaves have been suspected to possess *narcotic* properties, but some will not admit the fact: it was formerly kept in the shops as a *pectoral* and *narcotic*, but it is discarded from the present practice. Mr. Ray says, however, that Dr. Hulse used a decoction of the roots inwardly, and cataplasms of them outwardly, in *strumous* and *scrophulous* cases. Its scent is very disagreeable, and greatly resembles that of mice—*Withering.* Goats eat it; cows, horses, sheep and swine refuse it.

3. Symphytum. *S. officinale. S. consolida major. S. vulgare.*

*Ang.* Comfrey, common comfrey.

*Gen. Desc.* Bloss. funnel-shaped, bellying towards the top; mouth closed by hollow radiate valves, having an open hole on the outside near the border; nuts 4, perforated.

*Spec. Desc.* *Leaves* egg-shaped, decurrent. *cal.* close. *Bloss.* yellow-white; tube as long as the calyx; *valves* spear-shaped; flat, covering the anthers; edge studded with small shining glands. *River-banks, wet ditches.* Bloss. May.

There is a variety with red flowers.

*Use.* The root of this plant is a powerful *agglutinant*, good in the *fluor albus.*—*Hill.* The root is very mucilaginous; and being rather superior to the althæa, and more easily obtained, may be usefully substituted in its place, for for the general purposes of an *emollient* and *demulcent*. *Woodville*. Dr. Cullen says, there is no reason why, while mucilaginous matters are retained in our lists, symphytum should be omitted. It may be of service, as alleged, in *diarrhœas* and *dysenteries*. The roots are glutinous and mucilaginous: a decoction of them is used by dyers, to extract the colouring matter of gum lac. The leaves give a grateful flavour to cakes and panada; and the young stems and leaves are excellent when boiled. The particles of the *pollen* appear in the microscope like two globules united together. Cows and sheep eat it; horses, goats, and swine refuse it. *Linn.*

4. Borago. *B. officinalis. Buglossum latifolium.*
*Ang.* Common borage.
*Gen. Desc.* Bloss. wheel-shaped; mouth closed with rags; nuts 4, imperforated.
*Spec. Desc.* *Leaves* alternate, egg-spear-shaped, rough, as well as the stems, with white prickly hairs. *Bloss.* blue, white, or flesh colour, segments spear-shaped; *anthers* black; *filaments* above the insertion of the anthers, cylindrical, dark blue; below, thick, brown, glandular. *Walls, rubbish.* Bloss. June, August.
*Use.* The flowers have been termed *cordial,* and hence formerly much recommended in *melancholia,* and other affections of the nervous system. But as they possess neither pungency, warmth, nor fragrance, and as no saline matter appears to be contained in the flowers, any advantage supposed to be derived from a vinous infusion of them, can only be attributed to the menstruum. The leaves abound with a saltish juice, which on being boiled a sufficient time, forms crystals of nitre; hence the plant may be inferred to possess *refrigerating* and *aperient* virtues. *Woodville.*— The juice of this plant affords a true nitre. *See Experim. of M. Marggraff, Mem. de Berlin,* 1747, *p.* 72.—It is now seldom used internally, except as an ingredient in cool tankards for summer drinking; though the young and tender leaves are good in salads, or as a pot herb—*Withering.* A horse eat it.—*Dr. Stokes.*

5. Primula. *P. vulgaris. P. veris acaulis. P. sylvestris.*
*Ang.* Primrose.
*Gen. Desc.* Bloss. tube cylindrical, mouth open, stem within the tube; caps. 1-celled, cylindrical, many seeded, opening with ten teeth; summit a knob.
*Spec. Desc.* *Leaves* wrinkled, toothed; border of the blossom flat; leaf-stalks, when fully grown, longer than the leaves. *Woods, hedges, thickets, heaths.* Bloss. April, May.

*Use.* Of the roots, taken up in Autumn, and dried, a drachm and a half, according to Gerard, operates as a strong but safe *emetic* Silk-worms may be fed with the leaves of the primrose. *Trans. Soc. of Arts.* Sheep and goats eat it; cows are not fond of it; horses and swine refuse it. *Linn.*

6. Primula. *P. officinalis.*

*Ang.* Cowslip. Cowslip primrose. Pagils. Paigles.

*Gen. Desc.* *As above.*

*Spec. Desc.* *Leaves* wrinkled and toothed; *stalk* many-flowered. *Blossom* sweet scented, full yellow, an orange blotch at the base of each segment contracted at the middle of the tube, where the stamens are inserted; flowers drooping; border of blossom concave. *Meadows, pastures, in loamy or clayey soil.* Bloss. April, May.

*Use.* The leaves are sometimes eaten as a pot-herb and in salads. The blossoms are used for making cowslip wine. The root has a fine scent like anise—*Withering.* Silk-worms are fond of the leaves and flowers. *Trans. Soc. Arts.*

7. Meneanthes. *M. trifoliatum.* *Trifolium paludosum.* *Acopa.* *M. palustre tryphillum.* *Trifolium fibrinum.*

*Ang.* Marsh trefoil. Water trefoil. Marsh cleaver. Trefoil buckbean.

*Gen. Desc.* Bloss. hairy or fringed; nect. 5, at the base of the germen. Summit 2 lobed; caps. 1 celled.

*Spec. Desc.* *Leaves* by threes, spear-egg-shaped. *Bloss.* pinky and white, in a spike-like bunch, a floral leaf at the base of each pedicle; bloss. segm. entire at the edge, shaggy on the upper surface. One of the most beautiful of our native flowers. *In ponds and pits frequent.* Bloss. June, July.

*Use.* An infusion of the leaves is extremely bitter, and is prescribed in *rheumatisms* and *dropsies.* It is sometimes given to destroy *worms*—*Withering.* The blackness manifested by adding a solution of green vitriol to the juice, or to a strong infusion of the leaves, is a sufficient test of its *astringency*; while a drachm of the powdered leaves seldom fails to produce purging, or vomiting; so that in common with the tonic properties of a bitter, it seems also to possess a considerable share of medicinal activity. Its success in a number of chronic diseases is mentioned by various authors, as in *scurvy, dropsy, jaundice, asthma,*

*periodical*

*periodical head-achs, intermittents, hypochondriasis, cachexia, obstructio mensium, rheumatism, schrophula, worms, gout.* In gout, Dr. Boerhaave was relieved by drinking the juice mixed with whey: and Dr. Alston says, that this plant had remarkable effects in the gout, in keeping off paroxysms; though, he adds, not to the patient's advantage. Bergius confines the uses of this plant within narrower limits, and by later writers they have been still farther contracted. In Lewis's Mat. Med. (*by Dr. Aikin,*) it is said, "the leaves of buckbean have of late years come into common use as an *alterative* and aperient, in impurities of the humours, and some hydropic and rheumatic cases;" and as an active and eccoprotic bitter, we should suppose them not ill adapted to supply the want of bile in the *primæ viæ,* and thus infer their use in protracted jaundice and other biliary obstructions. Dr. Cullen had several instances of their good effects in some cutaneous diseases of the herpetic and seemingly cancerous kind. The leaves may be given in doses of ℈i. to ℈ij. two or three times a day, but a strong infusion of them is perhaps to be preferred, and with delicate stomachs it may be necessary to conjoin a grateful aromatic. These leaves impart their properties both to watery and spirituous menstrua—*Woodville.* This plant is regarded by the northern Europeans as a certain panacea for the cure of all diseases—*Ray.* The Highlanders in Scotland esteem a tea made of the leaves, as a good strengthener for a weak stomach—*Lightfoot.* In the north of Europe this plant has been used, in a scarcity of hops, to bitter ale instead of them, two ounces being equal to a pound of hops, so extremely bitter are they; yet Linnè observes, that in Lapland the poorer people make bread of the powdered roots mixed with meal, which, however, he acknowledges to be very unpalatable food. Some people smoke the dried leaves. As a proof of its good effects in dropsies, it has been said that sheep being forced to eat it, have been cured of the rot by it—*Woodville.* But it appears from the Upsal experiments, that sheep will seldom eat it; goats eat it; cows, horses, and swine refuse it—*Withering.*

8. ANAGALLIS. *A. arvensis.*

*Ang.* Pimpernel, Male pimpernel.

*Gen. Desc.* *Bloss.* wheel-shaped, caps. cut round, 1 cell, many seeds.

*Spec. Desc.* *Leaves* egg-spear-shaped. *Stem* trailing. *Calyx,* segments spear-shaped; *Bloss.* scarlet, sometimes blue. *Corn-fields, sandy places.* BLOSS. May, Aug.

*Use.*

*Use.* This plant is esteemed an *alexipharmic* and *sudorific*, but it is very little used. *Hill.*

9. CONVOLVULUS. *C. sepium.*

*Ang.* Great bindweed.

*Gen. Desc.* Bloss. bell-shaped, plaited. Nect. surrounding the base of the germen. Summits 2; caps. 2 or 3 celled, 2 seeds in each.

*Spec. Desc.* *Stem* twining. *Leaves* arrow-shaped, lopped at the base, edges brown. *Fruit-stalk* 4-cornered, 1 flower. *Bloss.* white; floral leaves 2, close to the cup. *Moist hedges.* BLOSS. July, Aug.

*Use.* The inspissated juice of this plant, in doses from 20 to 30 grs. is a powerful drastic *purge.* Scammony is the inspissated juice of the root of a foreign species of convolvulus, but so much resembling this, that they are with difficulty distinguished. Though an acrid purgative to the human race, it is eaten by hogs in large quantities without any detriment. Sheep, goats, and horses eat it; cows refuse it.—*Withering.*

10. CONVOLVULUS. *C. Soldanella.*

*Ang.* Scottish scurvy grass. Sea colewort. Sea bindweed.

*Gen. Desc.* *As above.*

*Spec. Desc.* *Stem* not twining, in open ground short, lying flat, and taking a semicircular direction; among bushes growing to some length embranched, bearing no flowers. *Leaves* kidney (sometimes heart) shaped. *Fruit-stalks* with 1 fl. *Bloss.* red. *Leafstalks* long. *Sea shore.* BLOSS. July.

N. B. It grows at a distance from the sea, but not above half its usual size.

*Use.* Of the juice, half an ounce, or a drachm of the powder, is an acrid *purge.* The leaves applied externally are said to diminish dropsical swellings of the feet.—*Withering.*

11. CAMPANULA. *C. rapunculus. Rapunculum.*

*Ang.* Rampions.

*Gen. Desc.* Bloss. bell-shaped. Filaments broad and arched at the base. Summit 3-cleft. caps. beneath; 3-celled, opening at 3 lateral holes.

*Spec. Desc.* *Leaves* waved, smooth, narrow; root-leaves spear-oval; *panicle* compact; fruit stalks by threes. *Stem* angular, rough, with milky juice. *Bloss.* large, purplish blue, nearly upright, not expanding; segments marked each with 3 red lines. *Hedge banks, fallow fields.* BLOSS. July, August.

*Use.* The roots are often eaten raw in salads, or boiled in the manner of asparagus. In gardens they are branched. The shoots of the c. latifolia, the beauty of whose flowers procures it a place in gardens, are boiled, the skin being stripped off, and eaten as greens about Kendal; it abounds also with milky liquor.—*Withering.*

12. Lonicera. *L. xylosteum.*

*Ang.* Upright honeysuckle.

*Gen. Desc.* Bloss. one petal, tubular, irregular; berry beneath, one to three-celled; many-seeded.

*Spec. Desc.* *Fruit-stalks* two-flowered, opposite, axillary. *Berries* distinct. *Leaves* very entire, pubescent, mostly egg-shaped, in opposite pairs, three pair on each branch, soft, and cloth-like to the touch. *Blossom* yellow, upper lip four-cleft, lower lip entire, strap-shaped. *Filaments* woolly. Shrub six or eight feet high. Bloss. May.

*Use.* Linnæus says this plant makes excellent garden hedges in a dry soil; and that the clear parts between the joints of the shoots are used in Sweden as tubes for tobacco pipes; the wood being very hard makes teeth for rakes, &c. It is rare, though certainly a native, in England.

13. Verbascum. *V. thapsus. V. album. Tapsus barbatus.*

*Ang.* Great white mullein. High taper. Cow's lungwort. Lady's fox glove.

*Gen. Desc.* Bloss. wheel shaped, nearly-regular; caps. two-celled, two-valved, many seeded.

*Spec. Desc.* *Leaves* decurrent, cottony on both sides. *Stem* unbranched, from four to six feet high. *Summits* globular. *Flowers* in a long terminating spike. *Blossom* yellow, rarely white. *Dry banks, chalky or gravelly soil.* Bloss. July.

*Use.* The leaves have an herbaceous, bittering, subastringent taste, but peculiar smell; on being chewed they discover a mucilaginous quality, and hence they are recommended as *emollients* both internally and externally; in the way of fomentation and cataplasm they are said to be an useful application to hæmorrhoidal tumors; and also for promoting the resolution or suppuration of glandular indurations; it has been recommended internally for catarrhal coughs and diarrhœas; Dr. Home found it successful only in the latter.—*Woodville.* In diminishing or stopping *diarrhœas* of old standing, or easing the pains of the intestines, Dr. Home found it useful; for the former purpose pose he advises a decoction, two ounces to a quart, of which he gave a quart every day, four ounces every three hours.—*Woodville.* It is used with advantage as an injection in tenesmus, and is often applied externally to the piles.—*Clin. Exp.* The flowers have also been used medicinally, being supposed to possess anodyne and pectoral virtues; but it is probable that no part of the plant deserves much consideration as a medicine.—*Woodville.* The seeds of this plant are said to intoxicate or stupify fish so that they suffer themselves to be taken out of the water with the hand.—*Bergius.* In the pulmonary complaints of cattle, it has been found of great service, and is used for consumptive cows in Norway. The down serves for tinder. Horses, cows, sheep, goats, and swine refuse it.—*Withering.*

14. Datura. *D. stramonium. Solanum fœtidum. Tatula. Ang.* Thorn apple.

*Gen. Desc.* Bloss. funnel-shaped, plaited; cal. tubular, angular, falling off with the blossom; caps. four-valved.

*Spec. Desc.* *Seed-vessel* thorny, upright, egg-shaped. *Leaves* egg-shaped, smooth, deeply indented. *Blossom* large, white, sometimes purplish. A large wide-spreading plant, native of America, but naturalized. *Rubbish, dung-hills.* Bloss. July.

*Use.* An ointment, prepared from the leaves, gives ease in external inflammations and *hæmorrhoides.* The Edinburgh College directs an extract to be prepared by evaporating the expressed juice of the leaves. This has been given with advantage in cases of convulsions, epilepsies, mania, especially that succeeding child-birth.—*See Woodville.* Out of fourteen epileptic patients at Stockholm, eight were cured, and five relieved, by Dr. Ohelius; the dose two to sixteen grains a day. This plant has been long known as a powerful *narcotic poison;* numerous instances are recorded of its deleterious effects, especially the seeds; which being taken internally have produced madness, tremors, swelling, itching, inflammation.—*See Med. Com. i.* 368, *iii.* 22. *Woodville, &c.* All parts of the plant possess a narcotic power, but the seeds are the only part of whose fatal effects instances are recorded; their soporiferous and intoxicating qualities are well known in the East, and are said to have been frequently converted to the most licentious and dishonorable purposes.—*Haller, Lindenstolphe.* By holding this plant to the nose for some time, or sleeping in a bed where the leaves are strewed, giddiness and stupor have been produced.—*Stork.* Externally the leaves of stramonium have been used as an application to inflammatory tumors and burns.—*Gerard.* Horses, cows, goats, and sheep refuse it.

15. Hyoscyamus. *H. niger. H. flavus. H. vulgaris. Ang.* Henbane. Black henbane.

*Gen. Desc.* Blossom funnel-shaped, blunt, irregular; stam. leaning; caps. with a lid, two-celled; seeds many, kidney-shaped.

*Spec. Desc.* *Leaves* embracing the stem, indented. *Flowers* sitting. *Blossom* tube white, middle deep purple, border pale yellowish brown, veined with purple; anthers and style deep purple. The whole plant woolly and clammy, with a strong peculiar odor. *Villages, road sides, rubbish.* Bloss. June.

*Use.* Henbane is a powerful narcotic poison, and many instances are recorded of its deleterious effects; from whence it appears, that any part of the plant taken in sufficient quantity is capable of producing the most alarming and terrible symptoms. See in *Phil. Trans. vol. xl. p.* 446, an account of the dreadful madness occasioned to nine persons from eating the root, attended with the remarkable circumstance that after their recovery for some days, all objects appeared bright red. See also for the effect of the seeds, *Phil. Trans. vol. xxxviii. p.* 99. And of the leaves boiled in broth,—*Ibid. vol.* 47. A few seeds of the henbane have been known to deprive a man of his reason and the use of his limbs.—*Lightfoot.* Dr. Smith however says, that he has often eaten the seeds with impunity.—*Withering.* Haller relates the contrary of a companion of his.—*Stirp. Helv. n.* 580. It is probable that the powerful qualities of this plant may under proper management be used with good effect in medicine; it was well known to the ancients and esteemed as an anodyne, but was laid aside till Baron Stoerck published cases of different diseases, in which extract prepared from the juice was found to be an efficacious remedy; such as internal spasms and convulsions, palpitations of the heart, madness, melancholy, epilepsy, inveterate head-achs, hæmoptysis. It has been found to produce sleep more powerfully than opium; and as it possesses a laxative quality, may be useful where the astringency of opium renders the use of it liable to objection. This medicine has not always been found equally successful, (Greeding, Cullen, &c.) but experience proves it to be an useful anodyne.—*Woodville.*

The

The leaves are said to have been applied with advantage externally, in the way of poultice, to resolve scirrhous tumors, and to remove some pains of the rheumatic and arthritic kind. The smell of the henbane is peculiar, and the bruised leaves emit an odor like that of tobacco; this is stronger if the leaves are burnt, and they sparkle with a deflagration resembling that of nitre, but to the taste they are mild and mucilaginous. It is poisonous to dogs and birds, but horses, cows, goats, and swine are not affected by it.—*Woodville.* Scattered about a house it is said to drive away mice. The Edinburgh College order the expressed juice to be evaporated to an extract, which may perhaps, in that state, be advantageously joined with opium, if costiveness is to be avoided; the dose is from half a scruple to half a drachm. Goats are not fond of it. Horses, cows, sheep, and swine refuse it, though sheep will sometimes eat it when young.—*Withering.*

16. Atropa. *A. belladonna. Belladonna trichotoma. Solanum lethale.*

*Ang.* Deadly nightshade. Dway berries. Deadly dwale.

*Gen. Desc.* Blossom bell-shaped; stam. distant; berry globular, two-celled.

*Spec. Desc.* *Stem* herbaceous, zigzag, two or three feet high. *Leaves* egg-shaped, entire. *Bloss.* dark purple with a yellow base, and greenish red outside. *Berry* green, changing to red, and black when ripe. *Hedges, among lime-stone and rubbish.* Bloss. June, August.

*Use.* This plant has long been known as a strong narcotic poison, and though the berries are less powerful than the leaves, we have many instances of their fatal effects, especially upon children, who have been allured by their beautiful appearance and sweet taste; they are recorded by a great variety of authors; and this is supposed to be the plant alluded to by Shakspeare, who makes Banquo say,

> "Or have we eaten of the insane root,
> Which takes the reason prisoner?" Macbeth.

For the dreadful symptoms produced, (vertigo, thirst, madness, convulsions, death,) see Dr. Woodville, l. c. Vinegar drank liberally has been found efficacious in obviating its effects, but evacuations should always be first promoted.—*Woodville.* The fresh leaves have been used externally with success to discuss tumors of the breasts of the scirrhous or even cancerous kind; and Dr. Graham says he found great benefit from a poultice made of the

 roots roots boiled in milk and applied to hard ill-conditioned tumors and ulcers.—*Med. Comm. vol. i. p.* 419. Their good effects in this way at length induced physicians to employ them internally for the same disorders; but though they have often been found a serviceable and important remedy, their success has not always been equal. Dr. Cullen however adduces several instances of its efficacy in the cure of cancer and scirrhosity. The sensible effects of the leaves, taken medicinally, are usually by the skin, the urinary passages, and sometimes by stool. Its operation is so uncertain that the proper dose can with difficulty be ascertained; six grains is a very large dose. Of the berries, successfully employed by Gesner, &c. as an anodyne in dysenteries, a small spoonful of a syrup of the juice was the dose given. The root has the same qualities, but less virulent—*Woodville.* It appears from a Case related by Ray, that where the skin is broken, the external application of the fresh leaf is attended with considerable danger, —*Ray Hist. Plant.* 680. A large glass of *warm vinegar* taken immediately after eating the berries will prevent any bad effect.—*Lightfoot.* The juice of the ripe berries stains paper of a beautiful and durable purple.—*Withering.*

17. Solanum. *S. dulcamara. S. scandens glycy-picros. Amaradulcis.*

*Ang.* Woody nightshade. Bitter-sweet.

*Gen. Desc.* Bloss. wheel-shaped. Anthers a little united, two holes at the top of each. Berry two-celled.

*Spec. Desc.* *Stems* smooth, rather shrub-like, zigzag, twining. *Leaves* egg spear shaped, the upper l. sometimes halberd shaped. *Flowers* in tuft-like bunches, purple, with two green spots at the base of each segment; sometimes flesh colour, rarely white. *Berries* two-celled, scarlet. *Moist brakes, hedges, sides of ditches.* Bloss. June, July.

*Use.* The roots and stalks, upon being chewed, first cause a sensation of bitterness, which is soon followed by a considerable degree of sweetness; hence the name of bitter-sweet. The berries have not yet been applied to the purposes of medicine, but they seem to act powerfully upon the primæ viæ, in exciting violent vomiting and purging; thirty were given to a dog, which soon became mad, and died in three hours, when, on his being opened, the berries were found in his stomach unchanged by digestion. The *stipites* or young twigs, are directed for use in the Edinburgh Pharm. either fresh or dried, allowance being made for the diminution of its powers in drying; they should be gathered in Autumn. The Dulcamara, though it it does not possess the narcotic qualities of other nightshades, is however admitted to be a medicine of considerable efficacy. Murray says that it promotes *all the secretions*, in which Bergius seems to coincide.—*Mat. Med.* 131. Haller observes, that it partakes of the milder powers of the nightshade joined to a resolvent and saponaceous quality. It has been recommended in diseases extremely various; Bergius confines it to *rheumatismus, et retentio mensium et lochiorum.* From the experiments of Razoux and others, it appears to have been used with advantage in some obstinate cutaneous eruptions.—*Woodville.* It is said by Boerhaave to be a medicine far superior to china and sarsaparilla as a sweetner and restorative. Linnæus says, an infusion of the young twigs is an admirable medicine in *acute rheumatisms, inflammations, fevers,* and *suppression of the lochia.* In *asthma* it was found very efficacious by Dr. Hill; and Dr. Hallenberg has advised it in *ischiatic* and *rheumatic* pains, *jaundice, scurvy,* and *lues venerea.* He (Linnæus also) directs a pint of boiling water to be poured upon two drachms of the stalks sliced and dried; after standing half an hour it must be boiled fifteen minutes; the dose is two tea cups full or more, morning and evening. The stalks may be gathered early in spring, but better in autumn, as the sensible qualities are then strongest.—*Med. Comm.* The root has the smell of the potatoe. —*Dr. Beddoes.* Sheep and goats eat it; horses, cows, and swine refuse it.

18. SOLANUM. *S. nigrum.*

*Ang.* Common nightshade. Garden nightshade.

*Gen. Desc. As above.*

*Spec. Desc. Stem* without prickles, herbaceous, branched, angular. *Leaves* egg-shaped, toothed, angular. *Bunches* nodding, pointing two ways. *Fruit-stalks* lateral, midway between the leaves. *Blossom* white. *Berries* black. This plant is subject to great varieties; with us it is herbaceous, in southern countries woody and very hard. *Rubbish, dunghills, kitchen gardens.*

*Use.* The leaves infused in boiling water, and taken at bed time, from one to three grains, occasion a copious *perspiration,* increase the *secretion by the kidneys,* and generally *purge* more or less the following day. These properties render it capable of doing essential service in various diseases, if judiciously applied, as may be seen in *Mr. Gattaker's Observations on the internal Use of Solanum.* But its effects on the nervous system are so considerable, and yet so uncertain, that it must ever be administered with the greatest caution. The leaves applied externally, abate inflammation and assuage pain.—*Withering.* This plant is faint and disagreeable to the smell, and possesses the deleterious qualities of the other nightshades in a very considerable degree, even its odor being so powerfully *narcotic* as to cause sleep. The berries are equally poisonous with the leaves, *(Wepfer De Cicut. p.* 226*)* and to poultry they are immediately fatal.—*Haller.* The leaves boiled, and eaten by a mother and four children, produced swellings of the face and limbs, followed by inflammation and gangrene, but the husband, who ate of it at the same time, found no consequent disorder. *(Rucker. Commerc. Noric.* 1731, *p.* 372*).* It is asserted by Mr. Bromfield, that in a dose of one grain it had a mortal effect upon one of his patients. It is however mentioned by Dioscorides, &c. as an esculent plant; and Guerin *(de Veget. ven. Alsatia, p.* 66) relates that he drank an infusion of fifteen grains without suffering; and that an epileptic patient took from half a drachm to two drachms of the expressed juice, without perceiving any narcotic symptom to follow; and a still larger dose, together with two drachms of the juice of the berries, being given to some soldiers, no other effect was produced than that of an increased quantity of urine. *(Murray, l. c.)* Externally it has been found useful as a *discutient* and *anodyne,* in various affections of the skin, tumefactions of the glands, ulcers, and disorders of the eyes; with the Arabians it is a common application to *burns* and *ulcers;* and Ray speaks highly of its effects in *indurations of the breast.* Mr. Gattaker has recommended, in a publication on the subject, its internal use in *old sores, scrophulous* and *cancerous ulcers, cutaneous eruptions,* and even in *dropsies;* one grain of the dried leaves, he says, infused in one ounce of water, sometimes produced considerable effect; in a dose of two or three grains it seldom failed to evacuate the first passages, or to increase sensibly the discharge by the skin or by the kidneys; and not unfrequently it occasioned head-ach, giddiness, dimness, and drowsiness.—*See Gat.* Mr. Bromfield however soon after declared, (see his *Account of English Nightshades*) that the cases in which he tried it, were much aggravated by it; and that its use is prejudicial and dangerous; Mr. G. renewed his assertion in *Essays on Medical Subjects,* in 1764, *(see Introduction and p.* 38*)—Woodville.* The flowers smell like musk. Horses, cows, sheep, goats, and swine refuse it.—*Withering.*

( To be continued. )

